# Clinical evidence deficiencies drive CHMP negative opinions and pre-opinion withdrawals in the EU centralized procedure (2021–2025)

**DOI:** 10.3389/fmed.2026.1875064

**Published:** 2026-07-17

**Authors:** Iris Pennings, Prerona Boruah, Alex Zwiers

**Affiliations:** Zwiers Regulatory Consultancy - A Product Life Group Company, Oss, Netherlands

**Keywords:** centralized procedure, CHMP, clinical efficacy, marketing authorization application, negative opinion, regulatory strategy, withdrawal

## Abstract

**Introduction:**

Market authorization applications that do not obtain authorization after CHMP review, whether through a formal negative opinion or pre-opinion withdrawal, remain poorly characterized at a systems level, despite substantial strategic, and commercial implications for applicants, and the broader impact on public-health.

**Material and Method:**

We conducted a retrospective descriptive analysis of 47 Article 8[3] new marketing authorization applications (MAAs) evaluated by the CHMP between 2021 and 2025, comprising 16 applications with a formal negative CHMP opinion and 31 withdrawn applications with an underlying negative benefit-risk assessment. Public assessment documents were reviewed, and documented CHMP concerns were scored across four predefined domains: CMC, non-clinical, clinical, and regulatory/procedural, including subsequent secondary sub-categorization to allow for more detail. Procedural timelines, re-examination outcomes, and multi-domain co-occurrence patterns were also summarized. Findings were summarized descriptively using counts, percentages, and procedural-duration comparisons.

**Result:**

Withdrawn and refused MAAs showed comparable product profiles and similar rates of orphan designation. Withdrawals occurred throughout all phases of the assessment procedure, reflecting different points at which applicants considered continuation no longer viable. Across both cohorts, procedural timelines frequently exceeded theoretical EMA benchmarks, with the excess concentrated in clock-stop phases. Clinical concerns were the most frequently documented domain, observed in 16/16 refused applications (100%) and 28/31 withdrawals (90%). Within the clinical domain, the secondary categories study-design concerns and insufficient evidence of efficacy were the most common findings and frequently co-occurred. Compared with refusals, withdrawals showed a broader deficiency profile, more often involving multiple domains, particularly CMC and regulatory/procedural concerns in addition to clinical deficiencies.

**Discussion:**

These observations suggest that unsuccessful outcomes in this cohort were commonly associated with limitations in evidence generation, dossier readiness, and alignment between regulatory pathway and evidence maturity. Earlier cross-domain readiness assessment may help applicants identify major unresolved deficiencies before or during CHMP review.

## Introduction

The European Medicines Agency's (EMA) Committee for Medicinal Products for Human Use (CHMP) is responsible for evaluating the benefit-risk profile of new medicines seeking marketing authorization (MA) through the European Unions' centralized procedure. When the CHMP concludes that the benefits demonstrated for a product do not outweigh its risks, it issues a negative opinion. This outcome has substantial implications for applicants, patients, and public health, because it can halt or substantially delay access to a medicine and may require major changes to the development or regulatory strategy (1).

Despite the importance of understanding why applications fail during CHMP review, unsuccessful marketing authorization applications (MAA) remain insufficiently characterized at a systems level. Published analyses have often focused on individual case studies or on specific product groups, such as advanced therapy medicinal products (ATMPs), orphan medicinal products, or subdivided by company size (2–4). Other studies have only examined decentralized procedures or combined decentralized and mutual recognition procedure datasets (5, 6). While these studies provide valuable insights, they do not fully characterize recent unsuccessful full-dossier applications submitted through the EMA centralized procedure, particularly when formal negative opinions and pre-opinion withdrawals are considered together.

In particular, it is important to distinguish between formal refusals and withdrawals in such evaluations, as they represent different endpoints, which may be associated with different underlying CHMP concerns. Within the centralized procedure, applications progress through defined assessment phases, including an initial and final assessment period, clock-stop phases during which applicants respond to CHMP questions, and, where applicable, a re-examination procedure following a negative opinion (7, 8). Alternatively, applicants may also withdraw an application before a formal CHMP opinion, with the extent of public information depending on the timing of withdrawal and the assessment documentation available (9). Although refusals and withdrawals therefore reflect different procedural decision points, both have outcomes in which unresolved concerns either prevented the CHMP from reaching a positive benefit–risk conclusion or influenced the applicant's decision not to continue the assessment process.

When evaluating the deficiency patterns underlying negative benefit–risk assessments, previous analyses have highlighted clinical data quality as a predominant driver, particularly insufficient evidence of efficacy and inadequate study design (10, 11). In addition, Chemistry, Manufacturing and Controls (CMC) deficiencies, regulatory and procedural non-compliance, and non-clinical data gaps have also been highlighted as main objections, though their relative contributions and co-occurrence patterns remain poorly characterized at a systems level (2, 3, 10, 11). Importantly, most published analyses are based on historical cohorts from more than a decade ago. Since then, the EMA assessment landscape has evolved considerably, with multiple guideline updates and further streamlining of regulatory procedures. Assessment of unsuccessful authorisations within a more recent timeframe may therefore provide additional insight into whether these changes have influenced the nature of objections underlying negative benefit–risk conclusions.

To date, no recent systems-level analysis has evaluated unsuccessful Article 8(3) full-dossier applications within the EMA centralized procedure by jointly evaluating formal negative CHMP opinions and pre-opinion withdrawals using a structured cross-domain framework. Consequently, it remains unclear how frequently deficiencies across the clinical, CMC, non-clinical, and regulatory/procedural concerns co-occur, whether withdrawn applications differ from refused applications in their overall objection profiles, or how factors such as timing of withdrawal or procedural prolongations are associated with these outcomes.

To address this gap, this study presents a retrospective descriptive analysis of Article 8(3) stand-alone, full-dossier MAAs evaluated by the CHMP between 2021 and 2025. The cohort included 47 non-authorized applications, comprising 16 MAAs that received a formal negative CHMP opinion and 31 applications withdrawn before an official CHMP opinion. Using a structured domain scoring framework across CMC, non-clinical, clinical, and regulatory/procedural categories, we characterized concern profiles in both groups, examined multi-domain co-occurrence patterns, and summarized procedural timing, including withdrawal phase, assessment duration, clock-stop prolongation, and re-examination outcomes. The findings aim to support applicants in identifying high-risk deficiencies earlier, strengthening submission readiness, and making more informed decisions on whether to continue review, withdraw an MAA, or pursue re-examination.

## Materials and methods

### Data source and study period

Initial selection of products was based on the Medicines Data Table from the EMA database, downloaded on 5 March 2026 and filtered for the period 2021–2025. For refused products, 2025 was applied as cut-off for the first CHMP opinion date; for withdrawn applications, the cut-off was applied to the date of withdrawal. Detailed product information was subsequently sourced from publicly available withdrawal and refusal European Public Assessment Reports (EPARs) and Questions and Answers (Q & A) documents published on the EMA website. Where submission dates were not explicitly stated in EPAR documentation, application dates were supplemented using information from Citeline's Pink Sheet, applicants' annual reports, or press releases.

### Inclusion and exclusion criteria

**Figure 1:**
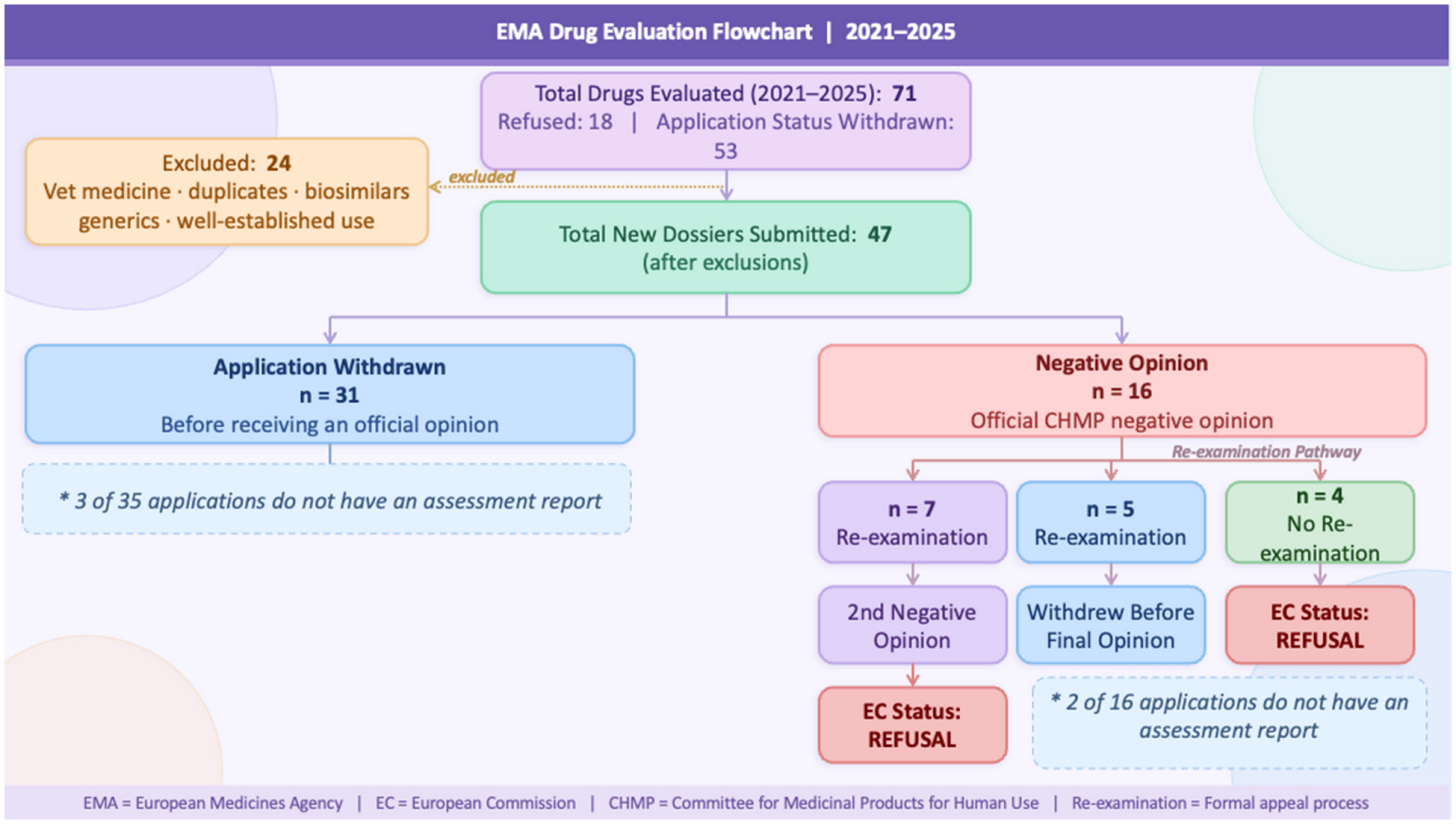
EMA drug evaluation flowchart, 2021–2025, showing excluded applications, withdrawn dossiers, negative CHMP opinions, re-examination pathways, and final EC refusal outcomes.

A total of 71 medicinal products with a negative opinion or withdrawn status were initially identified, including products that underwent re-examination (Figure 1). Of these, 24 were excluded based on predefined criteria: veterinary medicines, duplicate applications, biosimilars, generics, and well-established use applications. Exclusions were applied to ensure the cohort reflected only novel, full-dossier submissions under Article 8(3). The remaining 47 MAAs formed the final study population, comprising 16 that received a formal CHMP negative opinion (hereafter referred to as ‘Refused applications') and 31 that were withdrawn prior to an official opinion (hereafter referred to as ‘Withdrawals'). Of the refused applications, 12 applicants filed for re-examination, five withdrew during re-examination, seven received a second negative opinion, and four did not apply for re-examination. Of the 31 withdrawn applications, three did not have a publicly available assessment report, and 2 of the 16 refused applications lacked a detailed refusal assessment report; these were retained in the cohort where sufficient information existed but were excluded from secondary domain-level analyses where assessment data were required. The flowchart with product inclusion outcomes is shown in Figure 1.

### Domain scoring framework and product profile assessment

Each MAA was assessed using a structured framework of four predefined primary concern domains: CMC, non-clinical, clinical, and regulatory/procedural. These domains were selected to reflect the main dossier areas assessed during marketing authorization review and the structure of concerns commonly described in CHMP assessment documentation. Within each primary domain, secondary subcategories were applied to further characterize the nature of the identified deficiencies. The full secondary categorization scheme is provided in Supplementary Table 1.

For each application, public refusal or withdrawal assessment reports and Q & A documents were reviewed to determine whether concerns were present or absent within each primary domain and secondary subcategory. Each concern was scored as a binary variable at product level. For withdrawn applications, domains were primarily scored when they were listed as major objections (MO) in the withdrawal assessment report. For refused applications, domains were scored when they were described as contributing to the negative benefit-risk conclusion in the refusal assessment report or related Q & A documentation.

Applications could score in more than one primary domain and in multiple secondary subcategories; therefore, domain and subcategory totals are not mutually exclusive. Where a concern was mentioned only descriptively but was not linked to a major objection, withdrawal rationale, unresolved issue, or negative benefit-risk conclusion, it was not scored as a contributing concern. Accordingly, the analysis is based on the content and framing provided in the publicly available assessment reports, including how contributing factors to the benefit–risk assessment are documented and prioritized within these reports.

For products without a publicly available assessment report, domain-level scoring was only performed where sufficient information was available from Q & A documents or other public sources to support classification. Products with insufficient public information were retained in the overall cohort for descriptive procedural analysis where appropriate, but were excluded from domain-level analyses requiring detailed assessment information, and is consistently stated throughout the manuscript where applicable.

Each application was also categorized according to medicine class, therapeutic area, orphan designation status, and relevant regulatory pathway characteristics. Medicine class categories included small molecule, biological, ATMP, gene therapy, herbal product, and other product types where applicable.

### Estimation of EMA standard assessment times and classification of withdrawal phase

To compare the assessment timelines with the theoretical timelines of the EMA centralized procedure, benchmark durations were defined based on the Agency's procedural guidance (12). Because observed timelines in this study were calculated from the date of MAA submission to the date of withdrawal or CHMP opinion, the benchmark timelines also included the validation phase. The standard benchmark for a full procedure was therefore defined as approximately 349 days, comprising 19 days of validation, 210 days of active assessment, and two standard clock-stop periods of 90 days and 30 days, respectively. For re-examination procedures, a benchmark duration of 120 days was applied, comprising 60 days for submission of the detailed grounds for re-examination and 60 days for CHMP assessment.

Withdrawn MAAs were further categorized according to the phase at which withdrawal occurred and a corresponding theoretical benchmark duration was estimated. Classification was based on wording provided in the product-specific Q & A documents published on the EMA website:

During initial evaluation: “The application was withdrawn while the EMA was still evaluating the initial information from the company.” Benchmark duration: 139 days (13–19-day validation phase + Day 120).

During first clock-stop: “The application was withdrawn after the EMA had evaluated the initial information from the company and had prepared questions for the company. The company had not responded to the questions at the time of the withdrawal.” Benchmark duration: 229 days (13–19 + 120 + 90).

Between Day 121 and Day 180: “The Agency was assessing the company's responses to the questions at the time of the withdrawal.” This most likely reflects withdrawal after receipt of the joint assessment report around Day 157. Benchmark duration: 266 days (13–19 + 157 + 90).

During second clock-stop: “The company had not responded to the last round of questions at the time of the withdrawal.” Benchmark duration: 319 days (13–19 + 180 + 90 + 30).

Between Day 181 and Day 210: “After the Agency had assessed the company's responses to the last round of questions, there were still some unresolved issues.” This most likely reflects withdrawal after receipt of the draft joint assessment report around Day 195. Benchmark duration: 334 days (13–19 + 195 + 90 + 30).

During re-examination: “The company requested a re-examination of the Agency's recommendation, but it withdrew the application before this re-examination had finished.” Applications in this category were included in the refused group for subsequent analyses, as they had already received a negative CHMP opinion and were therefore evaluated against the benchmark timelines for refusal and re-examination.

### Data extraction, coding, quality control, and analysis

All product-level data were extracted into Microsoft Excel. Data extraction covered product characteristics, regulatory status, application and outcome dates, withdrawal phase, re-examination status, assessment duration, and the presence or absence of concern domains and subcategories. Two authors reviewed the assessment reports, extracted and curated data. Coding uncertainties were resolved by consensus, with senior author review where needed to ensure methodological and interpretive consistency. The coding framework was predefined before final data extraction and analysis, and was applied consistently across the full cohort. To support reproducibility, the secondary coding categories are provided in Supplementary Table 1, which allowed for consistent scoring and classification.

All analyses were descriptive, and no inferential statistical testing was performed. The aim of the study was to summarize how concerns are represented within publicly available regulatory assessment documentation of unsuccessful applications. Results are presented using counts, denominators, percentages, and procedural-duration summaries. Figures were generated using Microsoft Excel, PowerPoint, and BioRender biorender.com.

## Results

Across the 47 unsuccessful Article 8(3) applications, four main patterns were observed. First, withdrawn and refused applications had broadly comparable product profiles and orphan-designation rates. Second, withdrawals occurred across all phases of the assessment pathway, but most occurred after CHMP questions had been issued. Third, clinical concerns were the most consistently documented domain across both cohorts, with withdrawals additionally showing a broader multi-domain deficiency profile compared to refused applications. Fourth, both withdrawals and refusals frequently exceeded theoretical EMA assessment benchmarks, with delays concentrated in clock-stop periods.

### Product profiles of withdrawn and refused marketing authorization applications

Withdrawn and refused applications showed similar product-class distributions and broadly comparable rates of orphan designation (Supplementary Figure 1). Among the 31 withdrawn products, 18/31 (58%) were small molecules, 8/31 (26%) were biologicals, 2/31 (6%) were ATMPs, 1/31 (3%) was a gene therapy product, and 2/31 (6%) were classified as other product types, namely an allergen and irradiated cells/lysates. Overall, 13/31 withdrawn products (42%) had orphan designation, of which 9/13 (69%) were small molecules.

Among the 16 refused applications, 9/16 (56%) were small molecules, 4/16 (25%) were biologicals, 2/16 (13%) were ATMPs, and 1/16 (6%) was a herbal product. Overall, 8/16 refused products (50%) had orphan designation, of which 6/8 (75%) were small molecules. These findings indicate that the withdrawn and refused cohorts were not clearly separated by broad product class or orphan-designation status.

### Timing of withdrawal and procedural assessment duration

**Figure 2:**
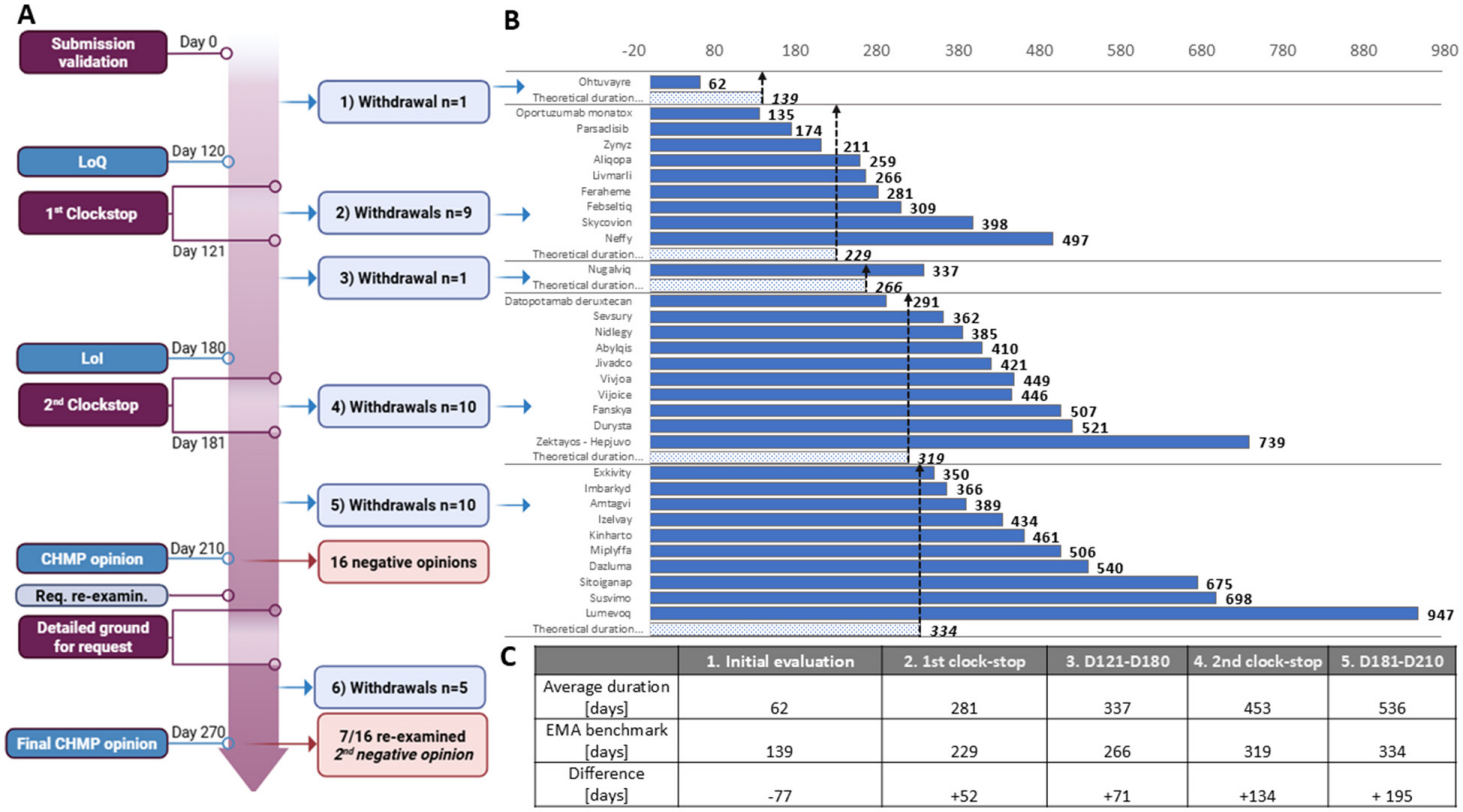
Comparison of the theoretical EMA/CHMP assessment timeline with the actual timing of withdrawals during the review process. **(A)** EMA centralized procedure assessment process, with key milestones and the number of withdrawals or negative opinions occurring at each stage. **(B)** MAA assessment duration upon withdrawal, in days for individual products, mapped onto the corresponding benchmarked EMA procedural duration. **(C)** Difference in procedure length, indicating delays from stage 2 onward (+).

Withdrawal decisions occurred at different stages of the CHMP assessment pathway (Figure 2A). One application was withdrawn before receiving the Day 120 list of questions (LoQ). No withdrawal assessment report was published for this product; however, press releases indicated that the withdrawal was driven by commercial considerations following company acquisition. Most withdrawals occurred after CHMP questions had been issued: nine applicants withdrew after receiving the LoQ without submitting responses, and one applicant withdrew after submitting responses to the LoQ. A further 20 applications were withdrawn after Day 180, either during or after the second clock-stop period; of these, 10 had not responded to the List of Outstanding Issues (LoI), while 10 withdrew after submitting responses.

Assessment durations generally exceeded the corresponding phase-specific theoretical benchmarks Figure 2B. The application withdrawn before Day 120 had an assessment duration of 62 days and remained within the expected first-assessment window. Among the nine applications withdrawn during the first clock-stop period without submitting responses, assessment durations ranged from 135 days to 497 days, and 6/9 exceeded the relevant benchmark, by a mean of 71 days. The application withdrawn after submitting LoQ responses but before Day 180 had an assessment duration of 337 days, corresponding to an excess of 90 days beyond its benchmark Figure 2C.

The longest delays were observed in applications withdrawn after Day 180. Among the 10 Day 180 non-responders, assessment durations ranged from 291 days to 739 days, with a mean duration of 453 days and a mean excess of 153 days. Among the 10 Day 180 responders who subsequently withdrew, durations ranged from 350 days to 947 days, with a mean duration of 536 days and a mean excess of 214 days. Thus, later-stage withdrawals were associated with progressively longer excess assessment durations.

### Concern domain profiles and multi-domain co-occurrence patterns in withdrawn applications

**Figure 3:**
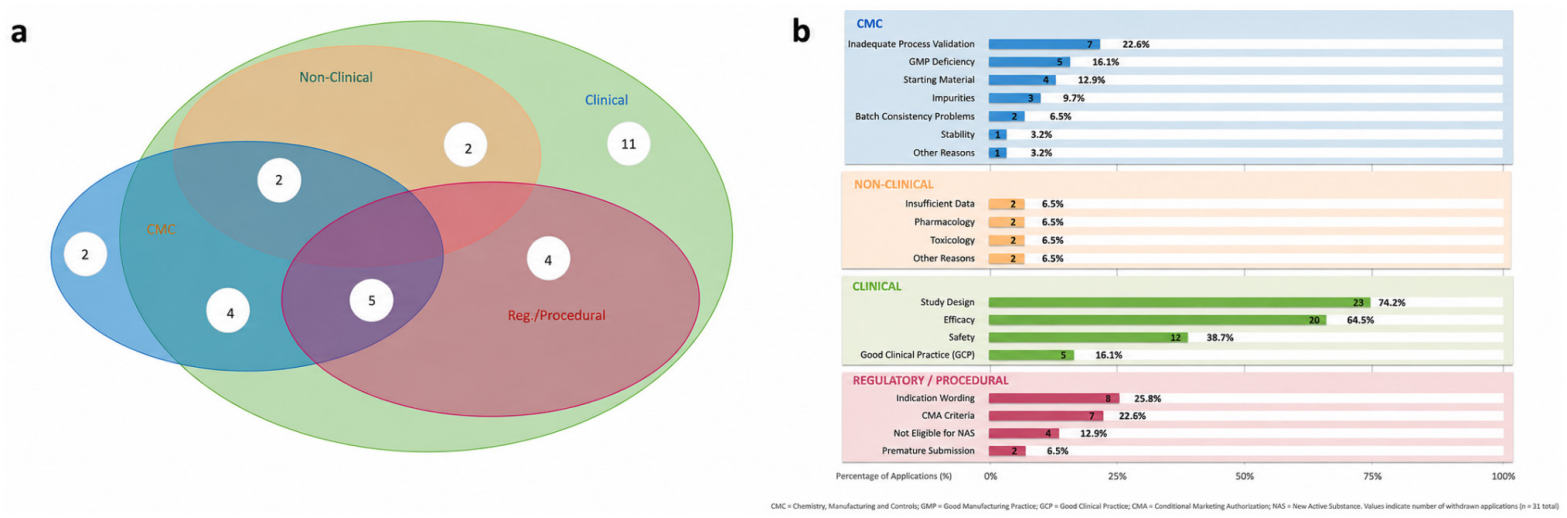
Concern domain profiles and multi-domain co-occurrence patterns in withdrawn applications (*n* = 31). **(A)** Venn diagram illustrating co-occurrence of concern domains. Numbers represent the count of applications per domain combination. One application did not score on any domain and is not included in the diagram. **(B)** Frequency of subcategory concerns within each primary domain, expressed as number and percentage of withdrawn applications (*n* = 31). Individual applications may contribute to multiple subcategories. CMC, chemistry, manufacturing and controls; GMP, good manufacturing practice; GCP, good clinical practice; CMA, conditional marketing authorisation; NAS, new active substance.

Reviewing the domains contributing to major objections (MOs), clinical concerns were the predominant driver of withdrawal, identified in 28/31 MAAs (90%). Study design deficiencies were the most frequently cited clinical subcategory, identified in 23/31 applications (74%), followed by insufficient evidence of efficacy in 20/31 applications (65%), safety concerns in 12/31 applications (39%), and GCP non-compliance in 5/31 applications (16%) (Figure 3B). Notably, the high rates of both study design and efficacy concerns within the same cohort suggest these frequently co-occurred within individual applications, potentially reflecting a pattern where flawed trial design directly undermined the basis for efficacy conclusions.

CMC deficiencies were identified in 15/31 applications (48%), with inadequate process validation being the most common subcategory, identified in 7/31 applications (23%), followed by GMP deficiencies in 5/31 applications (16%), starting material issues in 4/31 applications (13%), and impurities in 3/31 applications (10%). Regulatory and procedural concerns were present in 11/31 applications (35%), most commonly relating to indication wording in 8/31 applications (26%) and failure to meet conditional marketing authorization criteria in 7/31 applications (23%), with 4/31 applications (13%) deemed ineligible as a new active substance. Non-clinical concerns were the least frequently identified domain, affecting 4/31 applications (13%), with insufficient data, pharmacology, and toxicology each contributing across this small subgroup.

Domain co-occurrence analysis revealed that the majority of withdrawn applications were not characterized by isolated single-domain deficiencies (Figure 3A). Eleven applications carried concerns exclusively within the clinical domain. Two applications had CMC concerns alone, and a further two exhibited co-occurrences of CMC and non-clinical deficiencies. Five applications demonstrated co-occurrence of CMC and regulatory/procedural concerns alongside clinical concerns, while four applications presented with regulatory/procedural concerns as the sole identified domain along with clinical concerns. Two applications exhibited a three-way overlap across CMC, non-clinical, and clinical domains, and one application did not score on any domain as it was withdrawn prior to receiving the LoQ. These patterns indicate that multi-domain deficiency profiles were common, particularly the co-occurrence of clinical and regulatory/procedural concerns, suggesting that weaknesses in study design and evidence generation were often compounded by broader submission or eligibility issues.

### Procedural timing of refused applications

**Figure 4:**
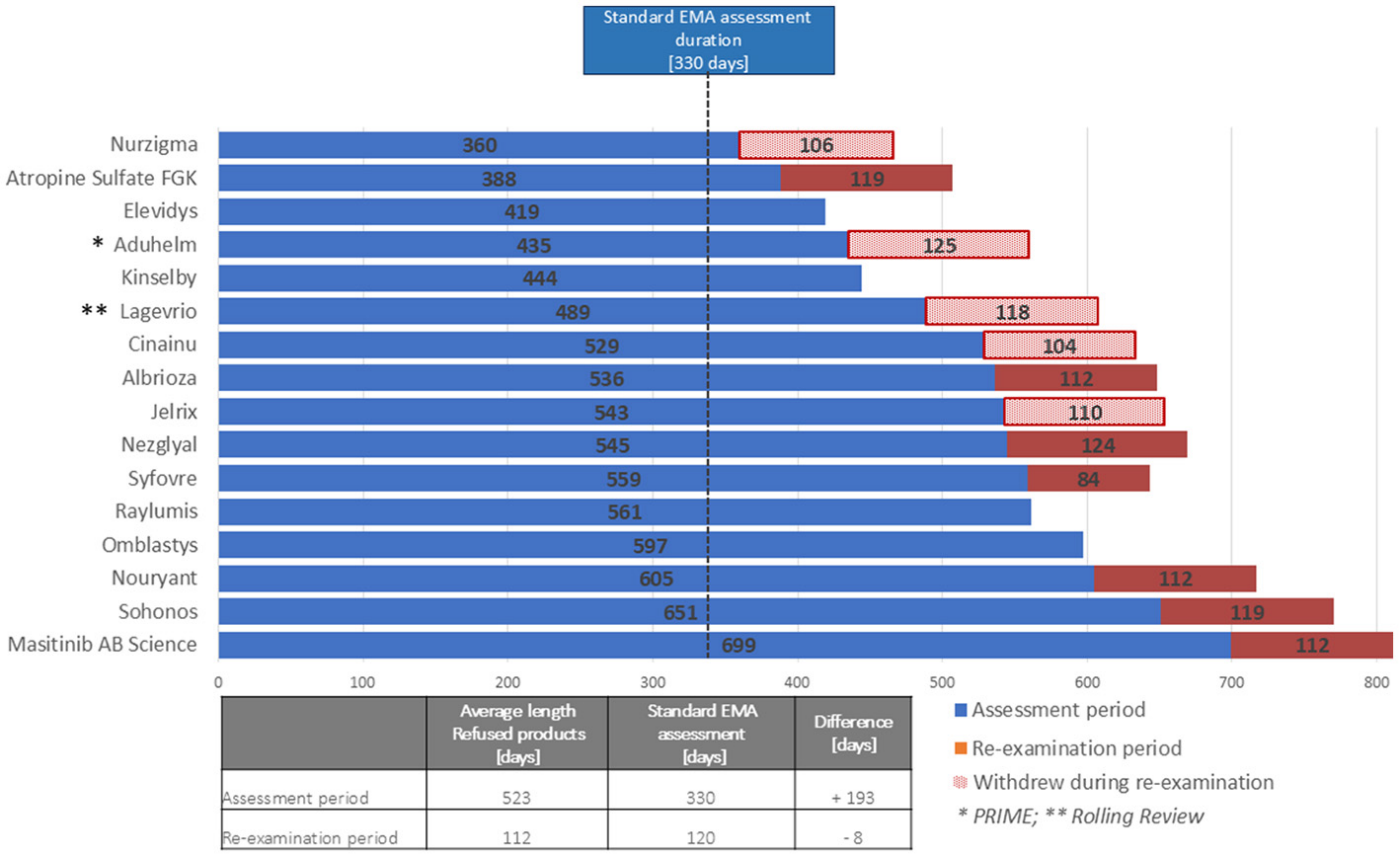
Duration of EMA assessment per MAA and re-examination periods. Assessment duration until official negative CHMP opinion, in days for individual products. Actual durations are mapped onto the corresponding benchmarked EMA period, with the difference in procedure length. If re-examination was requested, this is included for each individual product. *PRIME scheme review; **rolling review procedure.

All 16 refused applications exceeded the standard EMA assessment duration, with assessment periods ranging from 360 days to 699 days and a mean duration of 523 days (Figure 4). This represented a mean excess of 193 days beyond the theoretical benchmark. Two applications had undergone accelerated regulatory support or review mechanisms before submission: Aduhelm was reviewed through the PRIME scheme, and Lagevrio underwent rolling review for COVID-19. Both nevertheless exceeded the standard assessment timeline.

Of the 16 refused applications, 12 proceeded to re-examination. The mean re-examination duration was 112 days, which remained within the theoretical 120-day benchmark. Of these 12 applications, five were withdrawn during re-examination before a final opinion was issued, while seven received a second negative opinion. Four applications did not pursue re-examination, and their assessment periods ranged from 419 days to 561 days.

Further analysis of clock-stop periods indicated that the prolonged overall timelines were mainly attributable to clock-stop extensions rather than to the re-examination phase. For two products, Jelrix and Kinselby, clock-stop dates could not be retrieved because assessment reports were not available. Among the remaining applications, only six had a first clock-stop duration aligned with the predefined 90-day benchmark, and only four adhered to the predefined 30-day benchmark for the second clock-stop. One application, Masitinib AB Science, had a third clock-stop in addition to the rolling-review product. The mean clock-stop duration was 191 days, including the PRIME and rolling-review products.

### Concern domain profiles and multi-domain co-occurrence patterns in refused applications

**Figure 5:**
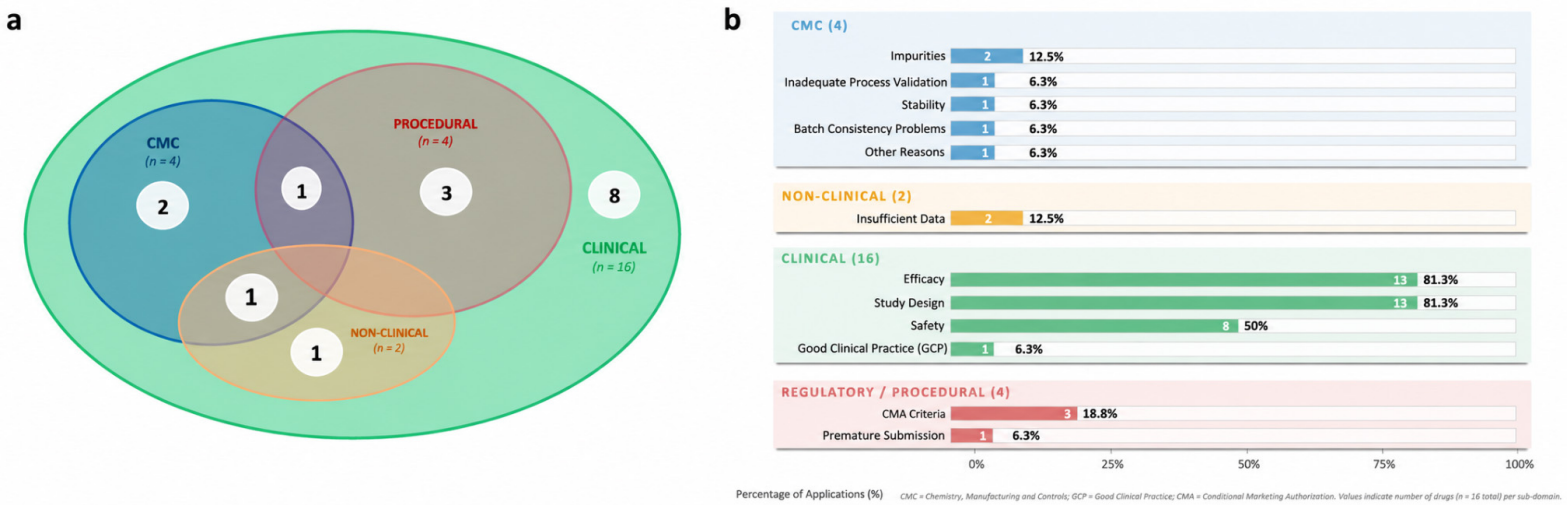
Concern domain profiles and multi-domain co-occurrence patterns in refused applications (*n* = 16). **(A)** Venn diagram illustrating co-occurrence of concern domains. Numbers represent the count of applications per domain or domain combination. Clinical concerns were identified in all 16 applications. **(B)** Frequency of subcategory concerns within each primary domain, expressed as number and percentage of refused applications (*n* = 16). Individual applications may contribute to multiple subcategories. CMC, chemistry, manufacturing and controls; GCP, good clinical practice; CMA, conditional marketing authorization.

Clinical concerns were identified in 16/16 refused applications (100%), with efficacy and study design deficiencies each present in 13/16 applications (81%), followed by safety concerns in 8/16 applications (50%) and GCP non-compliance in 1/16 application (6%) (Figure 5B). The equal frequency of efficacy and study design concerns across the cohort strongly suggests systematic co-occurrence within individual applications, consistent with the pattern observed in withdrawn applications.

CMC concerns were identified in 4/16 applications (25%), with impurities being the most frequently scored subcategory, identified in 2/16 applications (13%). Inadequate process validation, stability, and batch consistency were each identified in 1/16 application (6%). Non-clinical concerns were present in 2/16 applications (13%), with insufficient data as the sole subcategory identified. Regulatory and procedural concerns were present in 4/16 applications (25%), exclusively relating to failure to meet conditional marketing authorization criteria in 3/16 applications (19%).

Compared with withdrawals, refused applications showed a narrower co-occurrence profile, with fewer domains scored simultaneously (Figure 5A). Eight of 16 applications (8/16; 50%) carried clinical concerns as the sole identified domain. In applications with more than one scored domain, all additional concerns occurred in combination with clinical concerns rather than independently. These findings indicate that clinical concerns formed the central feature of formal refusals, while other domains generally acted as additional unresolved contributors.

### Detailed findings on drivers of withdrawals and negative CHMP opinions

The preceding sections show that clinical concerns were the most consistent findings in both refused and withdrawn applications. At the same time, withdrawals showed a broader domain profile and greater variation in procedural duration. The following subsections further depict the detailed results underlying these observations. First, the specific subcategories contributing to the overall clinical insufficiencies are shown, followed by analyses of the distinct subcategory drivers associated with withdrawals and refusals, including the timing of withdrawal and assessment timelines.

#### Clinical insufficiency as the main driver of negative and anticipated negative benefit-risk assessments

The dominance of clinical concerns in both cohorts is the most consistent finding in this dataset. Within this domain, the sub-categories study-design and insufficient evidence of efficacy were both frequently identified and often co-occurred within the same application, suggesting a mechanistic relationship rather than two independent failure modes.

Recurring objections in this domain included incorrect use of single-arm trials where a randomized controlled design would have been feasible, reliance on surrogate endpoints without established clinical relevance, *post-hoc* subgroup analyses, insufficient duration of follow-up relative to the disease course, and proposed indications that could not be translated into a testable hypothesis. Taken together, this suggests that the issue is less about ambiguous trial results and more about study design choices that restrict the evidence that can be generated to prove clinical efficacy. An additional finding in this study, not previously described in the literature, is the presence of GCP-related issues, identified in 5/31 withdrawals (16%) and 1/16 refusals (6%). While observed in a limited number of cases, this is notable, as GCP compliance forms a fundamental requirement for the acceptability of clinical data. Altogether, these MOs may reflect limitations in the development strategy rather than failure of the product itself. This interpretation is supported by previous studies, which have similarly highlighted the importance of the overall development plan in determining regulatory success (4, 11).

Safety concerns were also identified within the clinical domain as major objections, occurring in 12/31 withdrawn applications (39%) and 8/16 refused applications (50%). Across the reviewed public assessment documents, safety concerns included issues such as serious adverse events, off-target toxicity, hepatotoxicity, immunogenicity, reproductive risk, unknown long-term safety, excess mortality, and insufficient characterization of safety in the intended population.

Consistent findings related to clinical insufficiencies have been reported across previously published literature. Earlier studies, many of which evaluated EMA procedures from approximately a decade ago, also identified clinical objections, particularly inadequate evidence of efficacy and study-design limitations, as important contributors to unsuccessful marketing authorization applications (2–6). Although these historical cohorts are not directly comparable with the present 2021–2025 dataset because of differences in regulatory context, product mix, and assessment practice, they provide useful background suggesting that clinical evidence generation has remained a recurring challenge over time (10). Regnström et al. further found that compliance with CHMP scientific advice on pivotal-study design variables, including endpoint choice, comparator selection, and statistical methods, was associated with a higher likelihood of approval (11).

Despite developments and knowledge generation in the regulatory field over the years, including updated EMA and ICH guidance, the present findings suggest that the main drivers of unsuccessful applications have remained largely consistent. However, this does not necessarily indicate that all current products lack efficacy, but rather that applicants may not always generate or present evidence in a way that fully meets regulatory expectations at the time of marketing authorization application.

#### Drivers of withdrawal: a broader, more heterogeneous deficiency profile

Withdrawn MAAs differed from formal refusals in that their deficiency profile was more diffuse, with individual products often scoring across multiple domains simultaneously. A notable feature of this pattern was the higher prevalence of CMC deficiencies, observed in approximately half of the withdrawn applications, approximately double the rate seen in refusals. Regulatory and procedural concerns were also more frequently identified. Together, this suggests that these types of deficiencies may influence the decision to withdraw rather than continue the assessment, particularly when they cannot be resolved within the available timelines.

Within the CMC domain, the most frequently observed subcategories included inadequate process validation, starting material issues, and GMP deficiencies. These types of objections typically require additional manufacturing work, re-inspections, or new stability data, and therefore cannot realistically be addressed within the standard clock-stop periods. This is in contrasts with some clinical deficiencies, which may still be discussed or partially addressed during the procedure. Withdrawal in these cases allows applicants to resolve the issues outside the procedural timeline and, where appropriate, re-submit with a strengthened dossier. This likely explains why these deficiencies were less frequently observed in the refusal cohort.

Beyond these subcategories, the CMC “other” category captured additional relevant issues, including deficiencies in excipient selection, formulation robustness, and, for combination products, unresolved concerns related to the medical device component. These were typically linked to device design, human factors data, or compliance with the Medical Device Regulation (MDR) requirements under Article 117 of Regulation (EU) 2017/745 (13, 14). Although less frequently observed, excipient-related issues reflect the increasing scrutiny applied by the CHMP to novel excipients and complex formulations. In such cases, the impact on the overall product design may be substantial, making continuation within the ongoing procedure less viable and potentially driving the decision to withdraw.

Regulatory and procedural concerns were also commonly identified and are of particular interest, as they relate more to submission strategy than to the underlying science *per se*. Four main patterns dominate this domain:

#### Failure to meet conditional marketing authorization (CMA) criteria

CMA, governed by Article 14-a of Regulation (EC) No 726/2004 and Commission Regulation (EC) No 507/2006, requires that four criteria are met: a positive benefit–risk balance on the available data; a reasonable likelihood that the applicant will be able to provide comprehensive post-authorization data; fulfillment of an unmet medical need; and a judgment that the benefit of immediate availability outweighs the risk inherent in the fact that additional data are still required (15, 16). A major objection scored in this category indicates that one or more of these criteria were not fulfilled, most often the requirement for a positive benefit–risk balance. In general, scoring in this domain reflected a mismatch between the request for CMA and the maturity of the available evidence, rather than indicating that the product itself was inherently unviable. This aligns with findings from the EMA's ten-year review of CMA, which showed that unsuccessful applications often resulted from pursuing the CMA route without a sufficiently mature clinical data package or a clear unmet medical need justification (16).

#### Ineligibility as a new active substance (NAS)

The NAS status, determined by the CHMP in line with Directive 2001/83/EC and the related reflection paper (17), establishes whether a substance is sufficiently distinct from existing authorized substances in terms of molecular structure, therapeutic moiety, or the nature of its activity as it affects data exclusivity and, in some cases, eligibility for the centralized procedure itself. This can undermine the regulatory basis of the application and may contribute to the decision to withdraw.

#### Deficiencies in indication wording

Although this may initially appear as a drafting issue, ‘indication of wording' is a substantive regulatory matter with direct commercial implications. The indication description defines the target population and positions the product within the comparator landscape. In the products that scored in this domain, the proposed indications were often broader than the studied population, did not reflect key eligibility criteria from the pivotal trials, or were not supported by the population in which efficacy was demonstrated. While such issues can, in principle, be addressed by restricting the indication and has been reported to help overcome the initial concerns about Phase III efficacy results (4), this may significantly reduce the commercial potential of the product. As a result, applicants may choose to withdraw rather than proceed with a more limited label. This is supported by survey data from SMEs in DCP/MRP procedures, where 21% reported not marketing a product due to restrictions introduced during review (6).

#### Premature submission

In these cases, applications were submitted before the dossier was sufficiently mature, for example prior to completion of confirmatory analyses, resolution of GMP findings, or availability of key validation data. This again suggests that the issue lies not in the underlying science, but in the timing of submission. Premature submissions are typically driven by competitive pressure, investor-driven timelines, or through miscalibrated internal regulatory readiness assessments, and represent a preventable contributor to negative outcomes. This is consistent with previous observations that the maturity of the evidence package at submission is an important predictor of regulatory success (4, 11).

The broader multi-domain deficiency profile observed in withdrawals (Figure 3), particularly when compared to refusals, can be interpreted in two ways. On one hand, it may reflect the overall quality of the dossier, where the cumulative extent of deficiencies leads applicants to conclude that continuation is not viable, driving the decision to withdraw. On the other hand, it is important to consider the procedural context. A substantial proportion of withdrawals occurred without submission of responses to the LoQ or LoI, meaning that identified deficiencies remained unresolved. In contrast, refused applications underwent the full assessment cycle, allowing some initially raised concerns to be addressed before the final opinion. As a result, the broader deficiency profile observed in withdrawals may partly reflect the lack of invested time in resolving the objections, rather than a fundamentally more complex set of issues.

#### Withdrawal timing in relation to major objection profiles

Understanding whether the timing of withdrawal is linked to specific underlying drivers may provide insight into applicant decision-making. To explore this, a more detailed sub-analysis was performed, evaluating the objection profiles shown in Figure 3B, according to the phase at which the application was withdrawn.

A notable finding emerged for applications that were withdrawn immediately after receipt of the LoQ at Day 120, without submission of responses (*n* = 9). Specifically, 4 out of 7 inadequate process validation findings were concentrated in this group (4 out of 9 applicants), as well as 5 out of 8 instances related to indication of wording (5 out of 9 applicants). These types of objections are typically difficult to resolve within a standard clock-stop period, which likely contributed to applicants deciding to withdraw immediately after receiving the LoQ rather than investing further time and resources.

For applicants withdrawing after receiving the LoI at Day 180, including both responders and non-responders, the primary drivers remained within the clinical domain, particularly safety, efficacy, and study design. However, notably, 3/5 GMP-related findings in the withdrawal cohort were observed in applications that withdrew after responding to the LoI at Day 180, reflecting the timing of GMP inspection outcomes that run in parallel with the assessment procedure.

#### Drivers of negative CHMP opinions in refused applications: the clinical domain and residual quality issues

In the refused cohort, the clinical concerns were present in all cases, and other domains occurred only in combination with clinical issues. These additional domains therefore acted as co-occurring rather than independent drivers of the negative outcome. The overall frequency of CMC deficiencies was lower compared to the withdrawal cohort; however, the distribution of subcategories was distinct. Impurities were the most frequently cited issue, while process validation, stability, and batch consistency were each observed in only a limited number of applications. These are typically active substance–related quality issues and, in the CHMP assessment reports, were more often described as unresolved concerns rather than clear failures, persisting across multiple rounds of questions.

The CMC “other” category in this cohort can therefore be interpreted as unresolved active substance quality issues, including questions related to identity, purity profile, structural characterization, or the manufacturing control strategy of the drug substance that could not be adequately addressed during the procedure. This contrasts with the CMC “other” category observed in the withdrawal group. In refusals, these issues generally relate to the active substance and its manufacturing chain, whereas in withdrawals they more often relate to the drug product, its formulation, or an associated medical device.

In all refused applications in which regulatory or procedural concerns were identified, the applicant had failed to meet the criteria for CMA. In these cases, the clinical evidence submitted during assessment was considered insufficient by the CHMP to support the requested CMA pathway. This indicates that refusal was not only related to deficiencies in the data, but also to the fact that the available evidence did not satisfy the criteria for CMA.

#### Assessment of observed timelines in relation to regulatory benchmarks

For both withdrawn and refused applications, variability was observed in the overall assessment duration and consistently exceeded the EMA benchmark timelines. These benchmarks represent theoretical standard durations, and the assignment to specific phases was estimated, as the exact timing of withdrawal was not always explicitly reported. One withdrawn application, which was discontinued before Day 120 for commercial reasons following an acquisition, was excluded from this analysis due to lack of an official EPAR.

Overall, the assessment durations generally exceeded the corresponding benchmark timelines. This was observed across almost all withdrawn applications, with only a few exceptions in the first clock-stop phase (*n* = 3) and between Day 121–180 (*n* = 1). A similar pattern was seen for refused applications, where all initial assessment phases exceeded the standard EMA timelines, although the re-examination phase itself remained within the expected duration.

Assuming that EMA timelines were adhered to on the Agency side, these extensions are likely driven by prolonged clock-stop periods requested by applicants to address the extensive lists of objections. For withdrawn applications, this cannot be verified, as clock-stop durations are not publicly reported in withdrawal assessment reports. For refused applications, however, detailed timelines were available and further analysis indeed showed that all applicants consistently prolonged their clock-stop periods, in some cases even up to several months to almost a year.

This observation aligns with EMA-reported data, indicating that in 2023, 42% of applicants requested clock-stop extensions, with average durations of 205 days in 2022 and 198 days in 2023 (18). This is consistent with the average clock-stop duration of 191 days observed in this dataset over the past 5 years. To address these extended timelines, the EMA introduced a more stringent approach to clock-stop extensions in July 2024. Early signals of this reinforcement may already be reflected in the current dataset, where three products had a clock-stop after the guideline reinforcement (Atropine Sulfate FGK, Elevidus, and Nurzigma); these products showed a more limited extensions, particularly in the second clock-stop phase. Of note, extensions remain possible, but stricter justification is required; potentially, this guideline reinforcement may influence both applicant behavior and future withdrawal patterns.

While exceeding EMA benchmark timelines is not uncommon, the observed durations also appeared longer when compared to recently published assessment timelines for oncological products (19), suggesting a potential association between prolonged assessment and negative benefit–risk outcomes.

## Discussion

This study characterized concerns underlying unsuccessful Article 8(3) marketing authorization applications evaluated through the EMA centralized procedure between 2021 and 2025. Across the refused and withdrawn cohorts, clinical concerns were the most consistently documented domain, particularly study-design limitations and insufficient evidence of efficacy. Withdrawn applications showed a broader multi-domain concern profile, with more frequent CMC and regulatory/procedural concerns, whereas refused applications were more consistently centered on clinical concerns. Assessment timelines also frequently exceeded theoretical EMA benchmarks, with prolongations mainly associated with clock-stop periods. The following section considers factors that may influence applicants' decision-making during the assessment process, particularly the choice to withdraw or continue to a formal CHMP opinion.

### Factors influencing withdrawal vs. formal refusal

Insight into the factors driving withdrawal vs. continuation to a formal CHMP opinion may help clarify applicant strategies and considerations. In practice, this decision is influenced by a combination of scientific, strategic, and commercial considerations, many of which are not captured in publicly available data. While no single factor fully explains the decision, it is relevant to explore and hypothesize which considerations may influence this choice.

One key factor may be the reputational impact associated with a formal negative CHMP opinion announcement, which may affect investor perception, future submissions, and parallel regulatory procedures. While in contrast, a withdrawal can be framed more strategically, for example as a reprioritization or shift in company focus, allowing greater control over external communication.

At the same time, competitive dynamics may push applicants to continue despite a high risk of refusal, particularly in first-to-market scenarios. Differences in regulatory experience may also play a role, as more experienced applicants may better recognize when objections are unlikely to be resolved and choose to withdraw earlier, whereas others may continue due to lack of experience in interpreting CHMP feedback.

The type and level of public disclosure following each withdrawal or refusal may further influence decision-making. A formal CHMP opinion results in publication of a full (refusal) EPAR, both for the initial assessment and in re-examined cases. In contrast, withdrawals lead to less extensive assessment reports, while withdrawals during re-examination still result in publication of the refusal EPAR, but without details of the re-examination phase (9, 20). Of note, withdrawals before Day 120 do not result in public assessment reports, since no CHMP discussion has taken place at that stage. These differences in transparency may factor into the decision to withdraw or continue until an official CHMP opinion. Potentially, applicants may also continue the procedure, even when resolution of objections is unlikely, to influence the content and wording of the final published report.

Finally, prior investment plays an important role. Given the significant time and resources already invested until submission, some applicants may choose to continue the procedure rather than withdraw, particularly when additional studies or a new submission is not feasible. This may, for example, be more pronounced for small and medium-sized enterprises, which often have limited portfolios or fewer alternative development pathways to fall back to.

Taken together, the decision to withdraw or continue to a formal CHMP opinion is multifactorial and extends beyond scientific considerations alone and instead reflects a holistic decision to determine what outcome is best to protect the companies and asset's long-term value.

### Limitations

Several limitations should be considered when interpreting these findings. First, the analysis relies on publicly available EPARs and assessment reports, which represent a redacted and edited version of the CHMP's deliberations; not all factors underlying the final benefit–risk conclusion may be fully captured. Although a structured framework was applied to categorize deficiencies across domains, the assignment remained, to some extent, dependent on author interpretation. A small number of withdrawn and refused applications lacked publicly available assessment documentation and were either excluded from domain-level analysis or included only on the basis of high-level information from the Q & A publications.

Second, the scope of the dataset should be considered when interpreting the findings. Only Article 8(3) full-dossier applications were included; biosimilars, generics, and well-established-use applications were excluded by design, and the findings cannot be extrapolated to those pathways. Because the dataset is restricted to withdrawn and refused applications, the observed patterns cannot be benchmarked against the full cohort of applications submitted for the centralized procedure in the same period, which may affect the interpretation of product-level characteristics, such as orphan designation. Finally, the relatively small number of applications included in the analysis, and their division into separate withdrawal and refusal cohorts, limited the value of formal statistical comparisons. The findings should therefore be interpreted as descriptive patterns observed within the public regulatory record rather than as statistically validated predictors of regulatory outcome.

Third, the 2021–2025 window spans both the later phase of the COVID-19 pandemic and the period surrounding reinforcement of the EMA's 2019 guidance on clock-stop extensions, both of which may have influenced procedural behavior in ways not fully captured here.

## Conclusion

Across 47 Article 8(3) marketing authorization applications that failed during CHMP review between 2021 and 2025, clinical insufficiency, particularly the coupling of study-design limitations with inadequate demonstration of efficacy, was the most consistently observed concern in the public assessment record, for both refused and withdrawn applications. Withdrawn applications carried a distinctly broader deficiency profile, with contributions from CMC, including excipients, formulation, and device issues, and from regulatory/procedural concerns spanning CMA-criterion failures, NAS ineligibility, indication-wording deficiencies, and premature submission. Procedural durations of unsuccessful authorization substantially exceeded theoretical benchmarks, with the excess concentrated in clock-stop phases. This finding is particularly relevant in the context of EMA's ongoing initiative to reduce clock-stop extensions and may already be reflected in early signals of shorter extensions in selected 2025 procedures. For applicants, these findings highlight the need for rigorous internal readiness assessment across the domains identified in this study, with emphasis on a mature development plan and aligned regulatory strategy. For regulators, these findings suggest a need for review mechanisms that identify major unresolved deficiencies earlier, before applications progress through lengthy clock-stop cycles. Overall, the observed patterns indicate a persistent gap between CHMP regulatory expectations and some drug development and registration strategies used by applicants, consistent with historical findings previously reported in the literature.

## Data Availability

The original contributions presented in the study are included in the article/Supplementary material, further inquiries can be directed to the corresponding author.
