# Supplementary material for: Clinical evidence deficiencies drive CHMP negative opinions and pre-opinion withdrawals in the EU centralized procedure (2021–2025)
Source: Front Med (Lausanne). 2026 Jul 17;13:1875064. doi: 10.3389/fmed.2026.1875064 (PMC13424458; doi:10.3389/fmed.2026.1875064)
Supplement: Supplementary file 1 [file Table_1.docx]

Supplementary Material


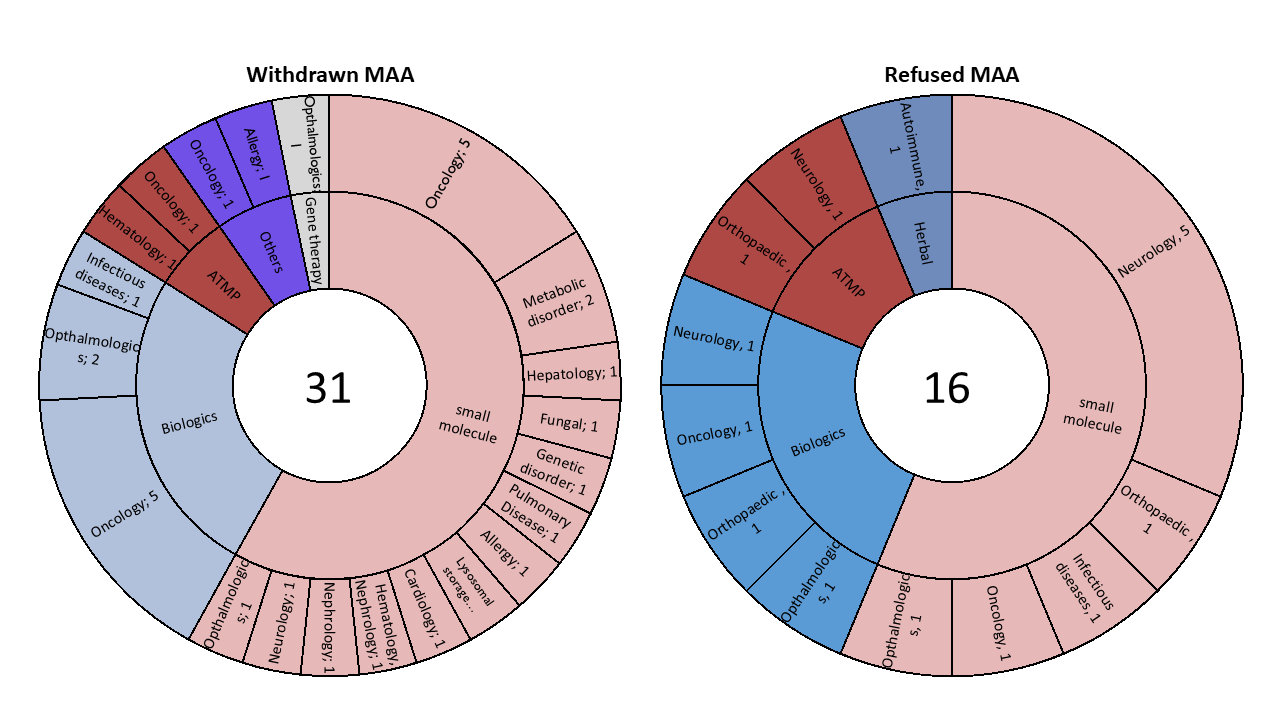


**Supplementary Figure 1. Product profiles of withdrawn and refused Market Authorization Applications. Sunburst chart show the amount of therapeutic area's in the outer ring per product class in the inner ring, relative to the total amount of withdrawn MAAs n=31 and refused MAA's n=16**


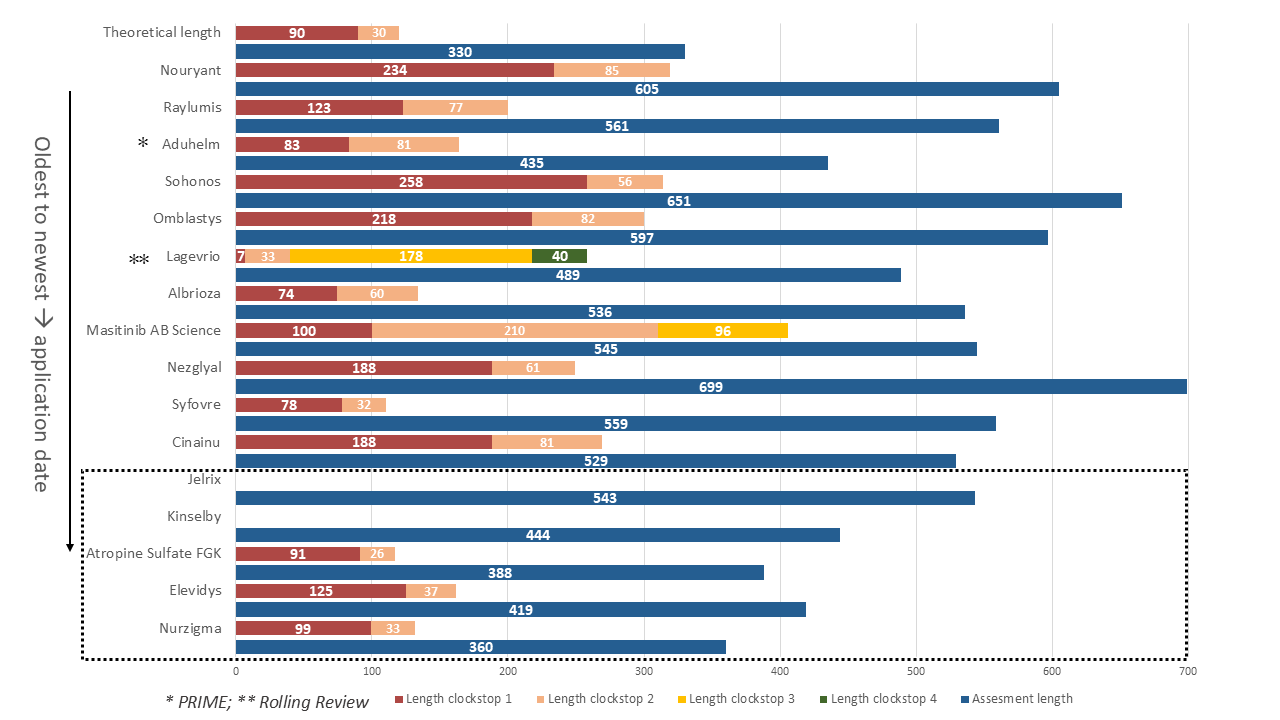


**Supplementary Figure 2. Clock-stop duration of refused Market Authorization Applications. Bar chart indicates the actual duration of the clock-stop and full assessment period, compared to the usual duration of the clock-stop period at EMA. Products are ordered from oldest application date to newest top to bottom. Dotted box represents the products which had had a clock-stop after the EMA guideline reinforcement in July 2024 (Atropine Sulphate FGK, Elevidus, and Nurzigma). For Jelrix and Kinselby no assessment report was available. * shows the PRIME scheme review and ** the product assessed via the rolling review procedure**

| Primary domain | Secondary category | Typical keywords for scoring |
| --- | --- | --- |
| CMC | GMP deficiencies | GMP non-compliance; inspection findings; audits required; non-compliance with EU GMP |
|  | Starting material |  |
|  | Stability | Stability data insufficient; shelf-life not justified or available |
|  | Inadequate process validation | process not sufficiently validated; lack of validation batches; manufacturing robustness not demonstrated |
|  | Batch consistency problems | Too much batch-to-batch variability |
|  | Impurities |  |
|  | Others |  |
| Non-clinical | Toxicology | reproductive toxicity; embryo-fetal toxicity; in vivo study toxicity; mutagenic impurities; unexpected metabolites |
|  | Insufficient data | Non-clinical package incomplete; absence of required in vivo data; absence of long-term toxicology/carcinogenicy data |
|  | Pharmacology | Mechanism of action not sufficiently characterised; off-target activity; lack of proof-of-concept data |
|  | Others |  |
| Clinical | Efficacy | Primary endpoint not met; small statistical difference; irrelevant effect size; effect not clinically meaningfull; contradictions across studies; dose finding not complete; futility |
|  | Study design | Unvalidated surrogate endpoint; incorrect endpoint; not sufficient power; non-representative study population; inappropriate comparator; small sample size; inadequate control arm; missing data; protocol deviations; changes in study protocol; relying on post-hoc analysis |
|  | Safety | SAEs; off-target toxicity; hepatotoxicity; immunogenicity; long-term safety unknown; reproductive risk; excess deceased |
|  | GCP non-compliance | Critical GCP findings; inspection deficiencies; data not reliable; informed consent issues; protocol deviations; inconsistent data management |
|  | Others |  |
| Regulatory / procedural issues | Not eligble as NAS |  |
|  | Premature submissions | Dossier incomplete; incomplete data package; data or study immature; ongoing study results pending |
|  | CMA criteria |  |
|  | No superiority to competitors | No added therapeutic value; no improvement over standard of care; lack of comparative advantage; too low efficacy to accept benefit-risk compared to competitors; no Major Therapeutic Advantage (MTA) |
|  | Others |  |
| Commercial reasons | Others | Business strategy; commercial reasons; change in applicant's strategy |

**Supplementary Table 1. Coding framework for classifying and scoring reasons for regulatory negative opinions or withdrawals. Primary domains, secondary categories, and representative keywords used to assign regulatory concerns to CMC, non-clinical, clinical, regulatory/procedural, or commercial reason categories. Keywords are illustrative and were used to guide consistent scoring.**
