# Supplementary figures and images for: Clinical evidence deficiencies drive CHMP negative opinions and pre-opinion withdrawals in the EU centralized procedure (2021–2025)
Source: Front Med (Lausanne). 2026 Jul 17;13:1875064. doi: 10.3389/fmed.2026.1875064 (PMC13424458; doi:10.3389/fmed.2026.1875064)

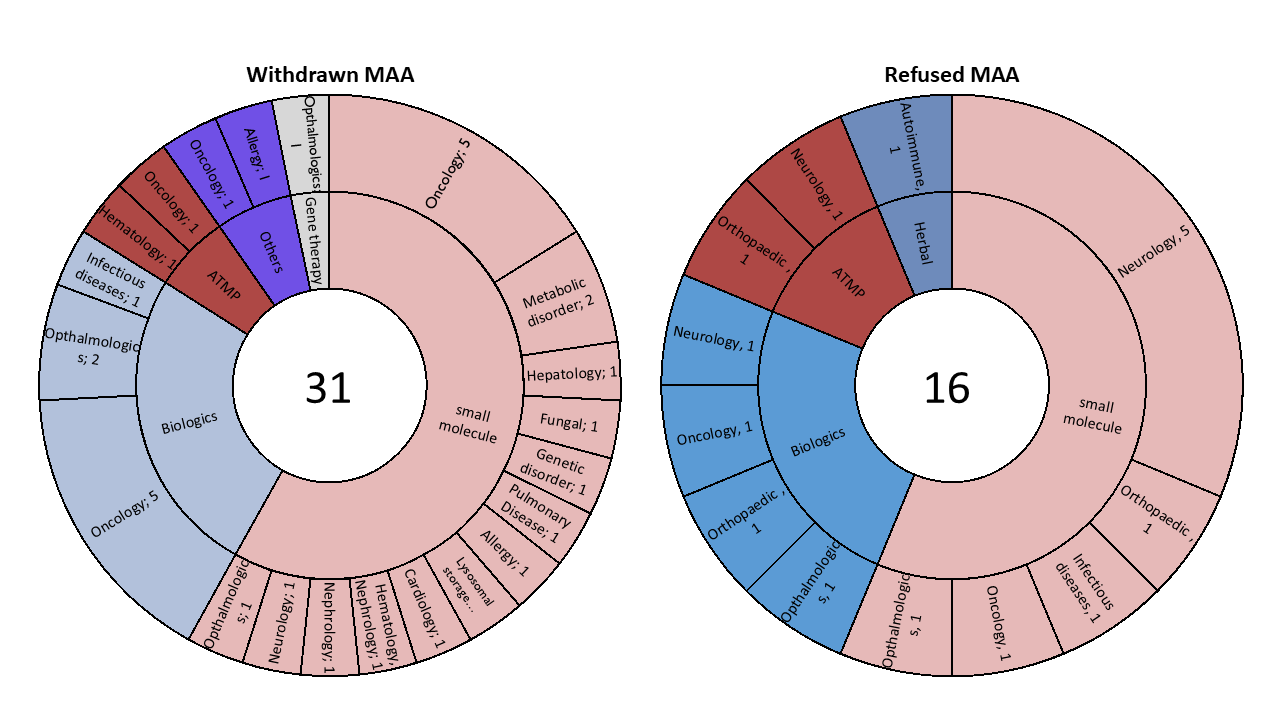

Supplement: Supplementary file 2 [file Image_1.tif]

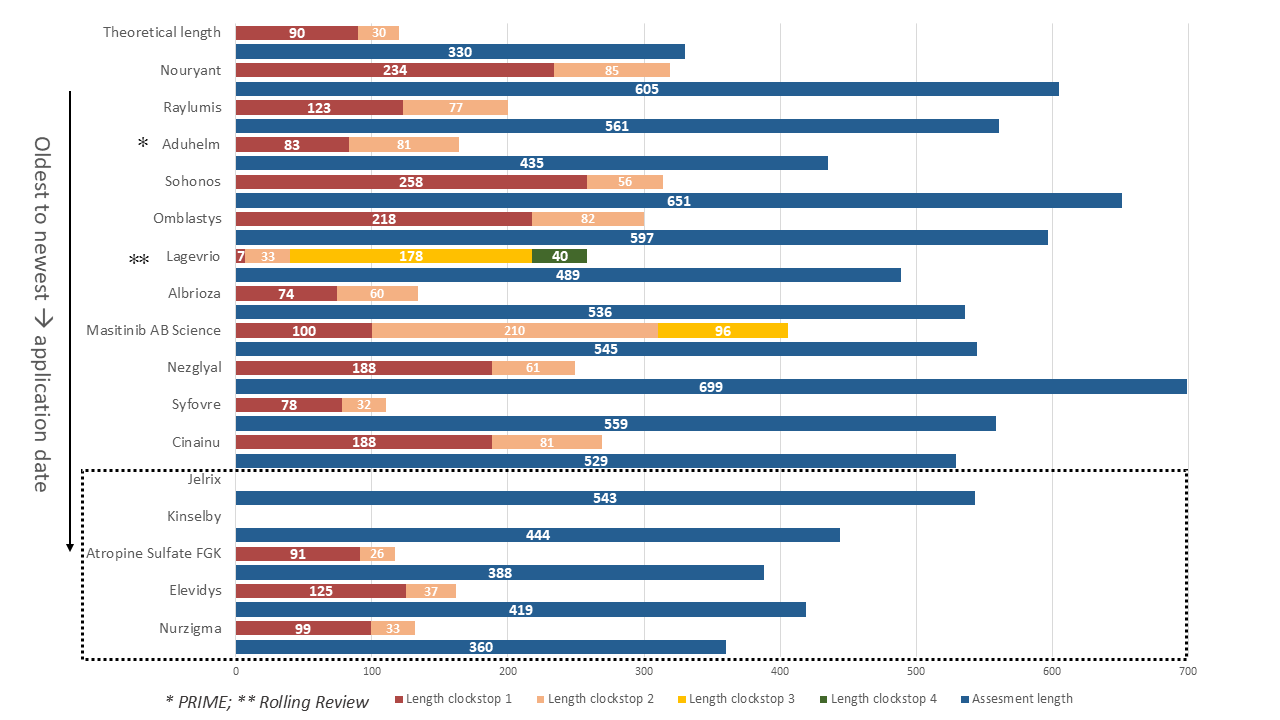

Supplement: Supplementary file 3 [file Image_2.tif]
